# Supplementary material for: Graphene Quantum Dots in Bacterial Cellulose Hydrogels for Visible Light-Activated Antibiofilm and Angiogenesis in Infection Management
Source: Int J Mol Sci. 2025 Jan 26;26(3):1053. doi: 10.3390/ijms26031053 (PMC11816408; doi:10.3390/ijms26031053)
Supplement: Supplementary file 1 [file ijms-26-01053-s001.zip › ijms-3420466-supplementary.pdf]

Supporting Information

Graphene Quantum Dots in Bacterial Cellulose Hydrogels for Visible Light-Activated Antibiofilm and Angiogenesis in Infection Management

Danica Z. Zmejkoski [1,*], Nemanja M. Zdravković [2], Dijana D. Mitić [3], Zoran M. Marković [1], Milica D. Budimir Filimonović [1], Dušan D. Milivojević [1] and Biljana M. Todorović Marković [1,*]

1 Vinča Institute of Nuclear Sciences—National Institute of the Republic of Serbia, University of Belgrade, P.O. Box 522, 11001 Belgrade, Serbia; zoranmarkovic@vin.bg.ac.rs (Z.M.M.); mickbudimir@gmail.com (M.D.B.F.); dusanm@vinca.rs (D.D.M.)
2 Scientific Institute of Veterinary Medicine of Serbia, Janisa Janulisa 14, 11107 Belgrade, Serbia; nemanja.zdravkovich@gmail.com
3 Faculty of Dental Medicine, University of Belgrade, Dr. Subotića 8, 11000 Belgrade, Serbia; dijana.trisic@stomf.bg.ac.rs
* Correspondence: danica@vinca.rs (D.Z.Z.); biljatod@vinca.rs (B.M.T.M.)

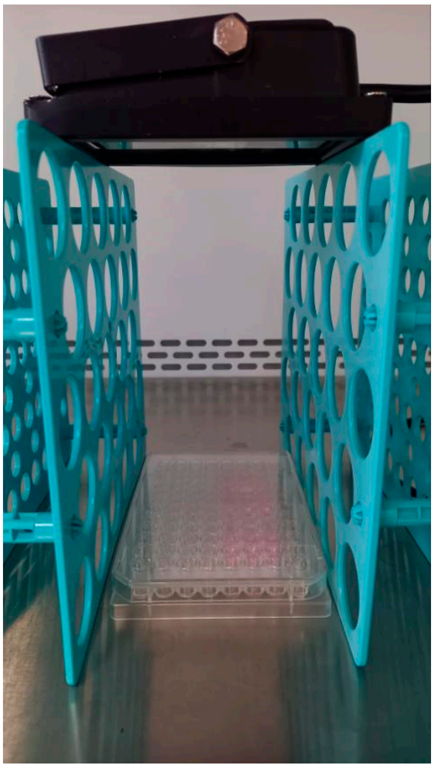

Figure S1. Experimental set-up for antibiofilm testing of BC-GQD composite hydrogel samples. To activate the photoactive effect, the plates were illuminated with green (537 nm) or blue light (470 nm) for 30 minutes, with the lamp of 3W positioned 20 cm away, with-out temperature changes near the samples and plates.

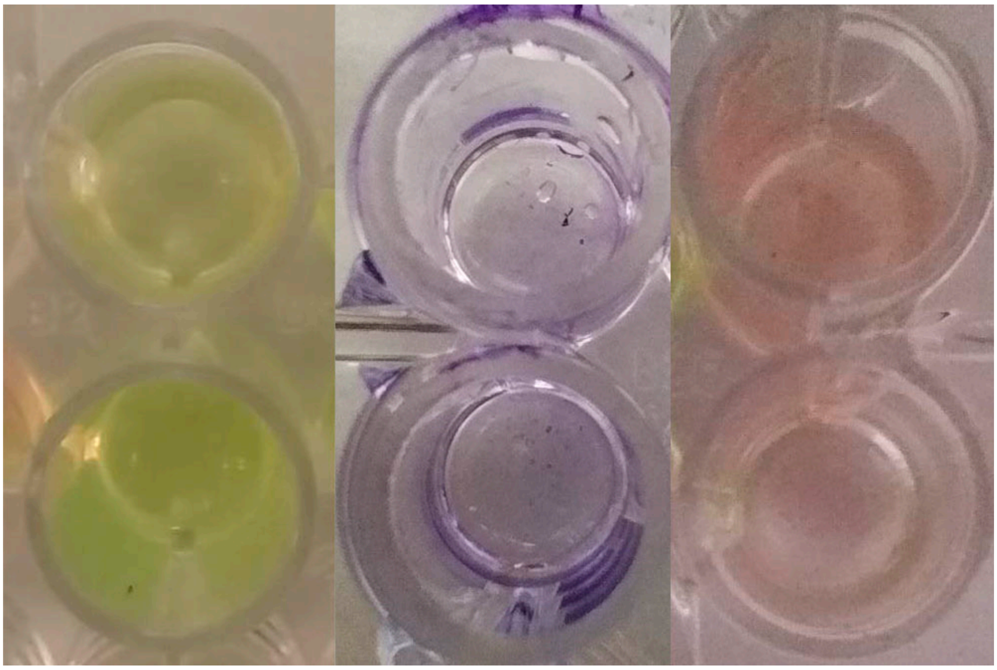

Figure S2. The growth of *P. aeruginosa* under BC-GQD (above) and BC (lower) hydrogels after blue light treatment. From left to right: growth before staining, after crystal violet and TTC stains. Vivid color intensity indicates lesser antimicrobial effect in control (BC).

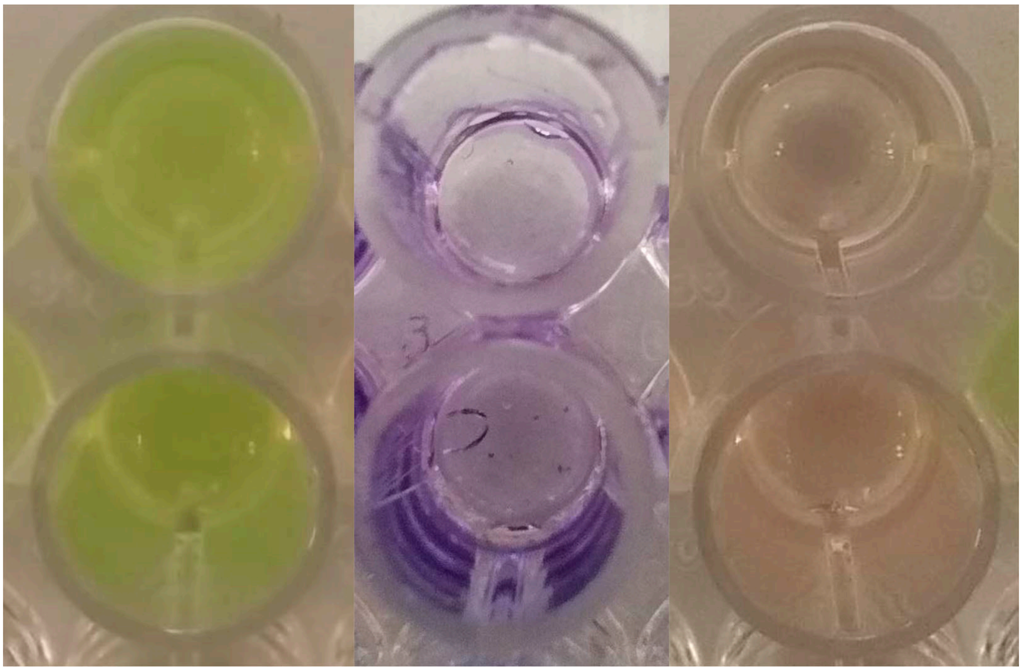

Figure S3. The growth of *P. aeruginosa* under BC-GQD (above) and BC (lower) hydrogels after green light treatment. From left to right: growth before staining, after crystal violet and TTC stains. Vivid color intensity indicates lesser antimicrobial effect in control (BC).

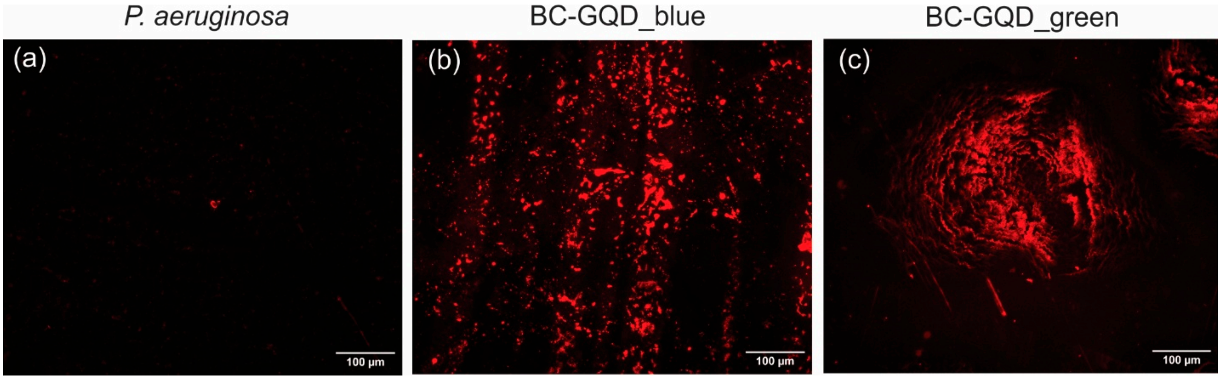

Figure S4. Fluorescence microscopy of biofilm of (a) *P. aeruginosa* (b) *P. aeruginosa* treated with BC-GQD_blue and (c) *P. aeruginosa* treated with BC-GQD_green, stained with propidium iodide. Scale bar was 100 µm.

The specificity of qPCR assays was confirmed by melting curve analysis. The qPCR efficiency of the assays for MMP9, eNOS, and Vimentin were calculated from standard curves derived from fivefold serial dilutions of the PCR products. The limit of detection (LOD) was determined by serial dilution of plasmid DNA containing the target sequence. The limit of quantification (LOQ) was determined by analyzing the precision (CV) and accuracy at low concentrations. All results are presented in Table S1.

Table S1. The specificity of qPCR assays. LOD – limit of detection; LOQ - limit of quantification.

**Parameter**	**MMP9**	**eNOS**	**Vimentin**
**PCR Efficiency (%)**	99.87%	99.46%	98.64%
**Slope**	-3.325	-3.335	-3.355
**R^2 value**	0.99	0.99	0.99
**Melting Peak (Tm)**	81°C	84°C	80°C
**LOD (copies/µL)**	15 copies/µL	12 copies/µL	18 copies/µL
**LOQ (copies/µL)**	54 copies/µL	52 copies/µL	58 copies/µL
**Precision (CV)**	<10%	<15%	<15%
